# Analyzing coastal dynamics by means of multi-sensor satellite imagery at the East Frisian Island of Langeoog, Germany

**DOI:** 10.1038/s41598-025-91306-3

**Published:** 2025-03-02

**Authors:** Julia Holzner, Günter Strunz, Sandro Martinis, Simon Plank

**Affiliations:** German Aerospace Center (DLR), German Remote Sensing Data Center, 82234 Oberpfaffenhofen, Germany

**Keywords:** multi-sensor remote sensing, coastal dynamics, sandy coast, meso-tidal coast, Geomorphology, Environmental sciences, Ocean sciences

## Abstract

Monitoring coastal dynamics is critical for the effective protection of coastal environments. Satellite remote sensing data offers significant potential to support this monitoring while also addressing the considerable challenges posed by the rapidly changing environmental conditions in coastal regions, such as tidal levels and currents. These challenges are particularly pronounced in meso- and macrotidal coastal areas. The goal of this study is to evaluate the effectiveness of a multi-sensor satellite remote sensing-based approach to assess coastal dynamics in a mesotidal environment, using the Island of Langeoog, Germany, as a case study. This approach also addresses the often limited availability of in-situ data in such regions. We employed high-resolution (HR) and medium-resolution (MR) optical data, alongside very high-resolution (VHR) Synthetic Aperture Radar (SAR) data, to detect coastal changes by analyzing several proxies, including the migration of sand bars, waterline position, dune toe location, and the extent of dry sandy coastal areas. To achieve this, we assessed and integrated thresholding and classification methods based on their suitability for specific sensors and proxies. Our findings demonstrate that combining different sensor types enables a more comprehensive analysis of various proxies of coastal dynamics. We successfully extracted instantaneous waterlines and identified migrating sand bars, linking these results to shoreline positions. Furthermore, our analysis revealed the direct influence of replenishment measures on beach conditions and suggested a stabilizing effect on the protective dune system. The findings display the uncertainties due to wave run-up and short-term variations in water level associated with analyzing dynamic meso-tidal sandy beach areas. Our results underscore the significant potential of multi-sensor data integration and diverse methodological approaches for supporting coastal protection authorities assessing the state of beaches.

## Introduction

About 30% of the world’s coasts that are not covered by ice are sandy coasts^1^. They represent highly dynamic landscapes, where changes are strongly influenced by multiple factors like meteorology, geology, marine processes, and human impacts^2^. As the interface between land and ocean, sandy coastal areas play a central role as natural coastal protection. Therefore, the understanding of accretion and erosion processes is crucial for monitoring sandy coasts and furthermore for an efficient implementation of coastal protection measures^3^. The main challenge in analyzing these processes is the high heterogeneity and dynamic of the coastal landscapes, especially in the case of the German Wadden Sea:^4^ it leads to widely differing demands on methodology, as well as spatial and temporal resolution to identify and analyze suitable proxies for the assessment of coastal dynamics.

When analyzing coastal dynamic patterns, various proxies can be utilized to characterize the state and evolution of coastal areas. The shoreline is often regarded as the key proxy for estimating the resulting coastal changes^5^. In a simplified approach, the shoreline is defined as the interface between land and water^6^. However, this idealized approach only considers the position of the shoreline at a specific point in time, addressing it as the instantaneous waterline. Rather, the instantaneous waterline is a highly time-dependent proxy that is subject to strong, short-term changes due to various processes, such as sediment transport and dynamic water level behavior caused by tidal range, waves, etc. This high variability has always to be taken into account when analyzing coastal processes. Two main groups of shoreline indicators are used to approximate the idealized shoreline:^7^ The first group contains indicators that can be identified based on visual features in aerial and satellite imagery (for example the instantaneous waterline). The second group is based on the dependence of the idealized shoreline on a specific tidal datum (for example the mean high tide (MHW)), and requires topographic information that can be obtained using in-situ surveys or LiDAR flights. However, relying solely on shoreline position and changes as a proxy has limitations when seeking a comprehensive understanding of coastal dynamics such as the underlying processes driving shoreline changes (e.g. sediment accretion due to onshore migration of sand bars) or the beach state (e.g. variations in sand deposit volumes or changes in beach slope). Especially in highly dynamic coastal environments, like the meso-tidal coast of the German Wadden Sea, the high-frequently changing position of the instantaneous waterline alone is not sufficient to provide reliable information on coastal change. It is therefore essential to incorporate additional proxies: Monitoring sand reefs and sand bars migrating onshore provides information about the island’s sediment budget, as the transported sand significantly contributes to the sand accretion processes of sandy beach areas. The term sand reef is used until the sediment body falls dry daily due to the tidal range and becomes a sand bar. If a sand bar joins the beach area of an island, this leads to a change in the position of the shoreline. Similar to shoreline definition, the exposed portion of a sand bar undergoes constant short-term fluctuations due to tidal levels and wave action. In contrast, its onshore migration can be identified as a mid-term process, occurring over months to years as the sand bar gradually emerges offshore and reaches the island’s coast. In addition to accretion processes and shoreline changes, the beach state offers crucial insights into coastal dynamics through various proxies, such as volume changes of the sand depot. When elevation data is unavailable, categorizing beach sections based on distinct patterns can serve as a useful alternative proxy to estimate changes of the sand depot. Therefore, the sandy beach areas can be divided into two categories: 1) Seaward lies the beach area with wet sand. It is located in the active breakwater zone and is occasionally flooded depending on tidal range and wave action; 2) The dry sand follows towards the landward side: these are beach zones that remain permanently dry under normal tides. Dry sandy beach areas undergo mid- to long-term variations driven by continuous erosion and accretion processes and therefore are suitable for monitoring on temporal scales from months to years. Landwards, the beach is often limited by a dune wall, with the bottom edge on the seaward side called the dune toe. As dunes act as a dynamic natural barrier against hazards such as storm surges^8^, analyzing their state and changes as an additional proxy for coastal dynamics provides valuable information for coastal protection measures. Since coastal dunes are less influenced directly by tidal levels and wave action - factors that strongly affect other mentioned proxies - their estimated changes typically occur over relatively longer timescales of months to years.

In our work, we focused on the described proxies to investigate the suitability of multi-sensor satellite remote sensing data for the development of an analytical approach that enables the comprehensive monitoring of coastal dynamics in higly dynamic meso-tidal sandy coastal environments.

### Satellite remote sensing imagery for analyzing coastal dynamics

Satellite-based remote sensing offers a number of advantages in terms of monitoring and analyzing coastal dynamics. In many cases, the monitoring of coastal zones is based on time-consuming and cost-intensive aerial and in-situ surveying campaigns^9–11^, which often depict small study areas or a sparse temporal resolution. The use of satellite remote sensing data overcomes these spatial and temporal limitations and has the capability to minimize the cost of monitoring^12^. The increasing availability and the constantly improving spatial and temporal resolution of satellite data in recent years are key factors here. The analysis of remote sensing data thus offers a reliable opportunity for the assessment of long time series of coastal dynamics. Research often uses medium (MR) and high (HR) resolution optical imagery, several studies evaluated the suitability of SAR (Synthetic Aperture Radar) data for that purpose, too. In terms of MR optical satellite data, two comprehensive databases, NASA’s Landsat archive and ESA’s Sentinel-2 archive as publicly available collections of remote sensing imagery facilitate the broad use of such data in the context of coastal dynamics^13,14^. However, many studies focus on single components of coastal processes, e.g. waterline and subsequent shoreline changes^15,16^, often in spatially extensive contexts^17,18^. The interactions between these components or an spatially high resolution analysis of the dynamics are rarely dealt with , especially in meso-tidal coastal environments such as the German Wadden Sea. But with these coastal landscapes representing highly dynamic environments comming with a wide variability regarding their geographical, geological and marine characteristics, there is a need for small-scale approaches regarding coastal dynamics to understand the fundamental processes leading to changes, especially for the realization of coastal protection measures. Therefore, HR optical satellite data are very suitable for a spatially detailed analysis^19,20^. Constellations such as PlanetScope^21^ provide a high temporal resolution with more than 200 active CubeSats currently in orbit. Limitations exist regarding data accessibility, as HR satellites are often commercial missions. Furthermore, optical sensors in general deal with constraints regarding the need for daylight and cloud-free weather conditions. SAR data overcomes these limitations by actively emitting radar pulses that hit the Earth’s surface, which are reflected and then detected by the sensor. Especially in the case of coastal monitoring, where frequent cloud cover often makes it difficult to acquire useful optical data, the evaluation of SAR data is a valuable and frequently used alternative to extracting the instantaneous waterline^22,23^. When processing data in the research field of coastal dynamics, statistical methods, based on classification or thresholding techniques, are popular approaches. In terms of optical data, the spectral information of the channels covering the visible electromagnetic spectrum (VIS) and additional NIR (Near-Infrared) information fulfill the requirements to identify the land-water boundary as well as to differentiate between land cover types, e.g. water, sand areas, vegetated land, etc. For a more detailed approach, often additional information extracted from several spectral band indices is used^20,24^. As the possibility of calculating specific indices depends on the channel variability of the specific sensors, the wide spectral coverage of Landsat (up to 11 bands) and Sentinel-2 (13 bands) is a particular advantage here. Due to the lack of spectral information in the SWIR (Short-Wave Infrared) regarding several HR sensors (e.g. PlanetScope, Pléiades), the availability of indices in the processing of HR optical data is often limited. In terms of SAR data, most studies focus on extracting the instanteous waterline by applying thresholding and filtering methods^25,26^. In addition to methodological developments, for all sensor types applications with a high degree of automation are becoming increasingly important^27,28^.

A multi-sensor approach appears promising in the context of a remote sensing-based investigation of coastal dynamics. By combining HR and MR optical and VHR SAR data, we aim to overcome the temporal, spatial, and spectral limitations of the single sensors. For that purpose, in this study we investigate the advantages and limitations of different sensor types and methods regarding several proxies for coastal dynamics to provide a multi-sensor data approach for the assessment of coastal dynamics. Data and methods are described in detail in the *Material and Methods* Section. We performed our analysis on the Island of Langeoog, which is described in the next section.

### Coastal dynamics at the Island of Langeoog, East Frisian Islands, Germany

**Fig. 1:**
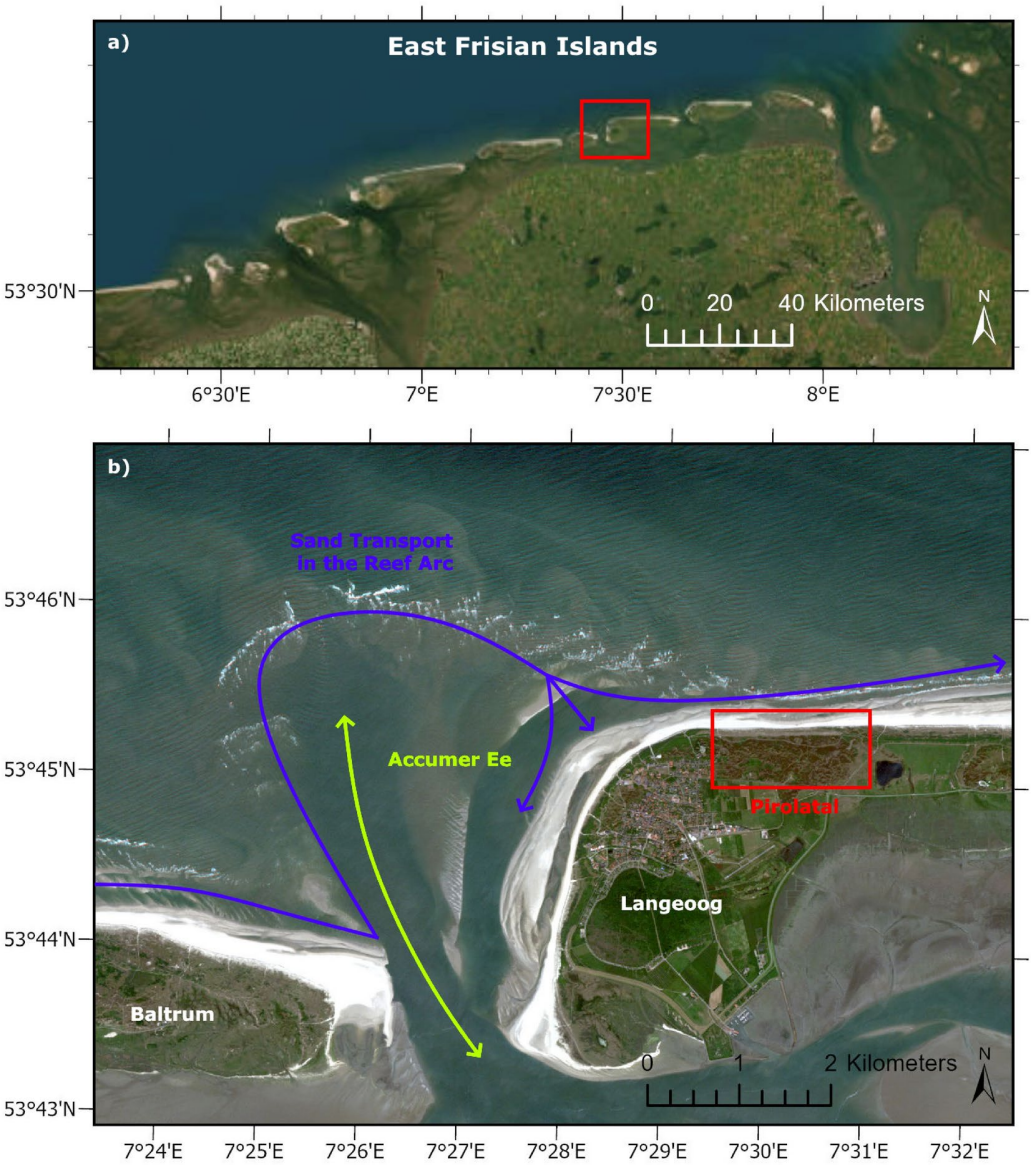
Overview map of the East Frisian Barrier Islands and the North Sea Coast. The research area is highligthed with a red box (**a**). Overview map of the western part of the Island of Langeoog. The natural sand supply of the island is based on the sediment transport running from west to east (purple). The tidal inlets between the islands (green) create so-called reef arches, in which the sediments from the east are first transported seaward and then, due to the ebb current, align themselves inland again and thus transport sediment onshore. Due to the particularly tight nature of the reef arc off the Island of Langeoog, this dynamic leads to the sediments migrating onshore at the north-western tip of the island and at the same time to ongoing erosion in the western coastal section off the Pirolatal (red) (**b**). Basemap: RapidEye (acquisitioned on June 11, 2015) (Source: 2015 Blackbridge AG, Germany). Map created using ArcGIS Pro 3.2.0 (https://www.esri.com/).

**Fig. 2:**
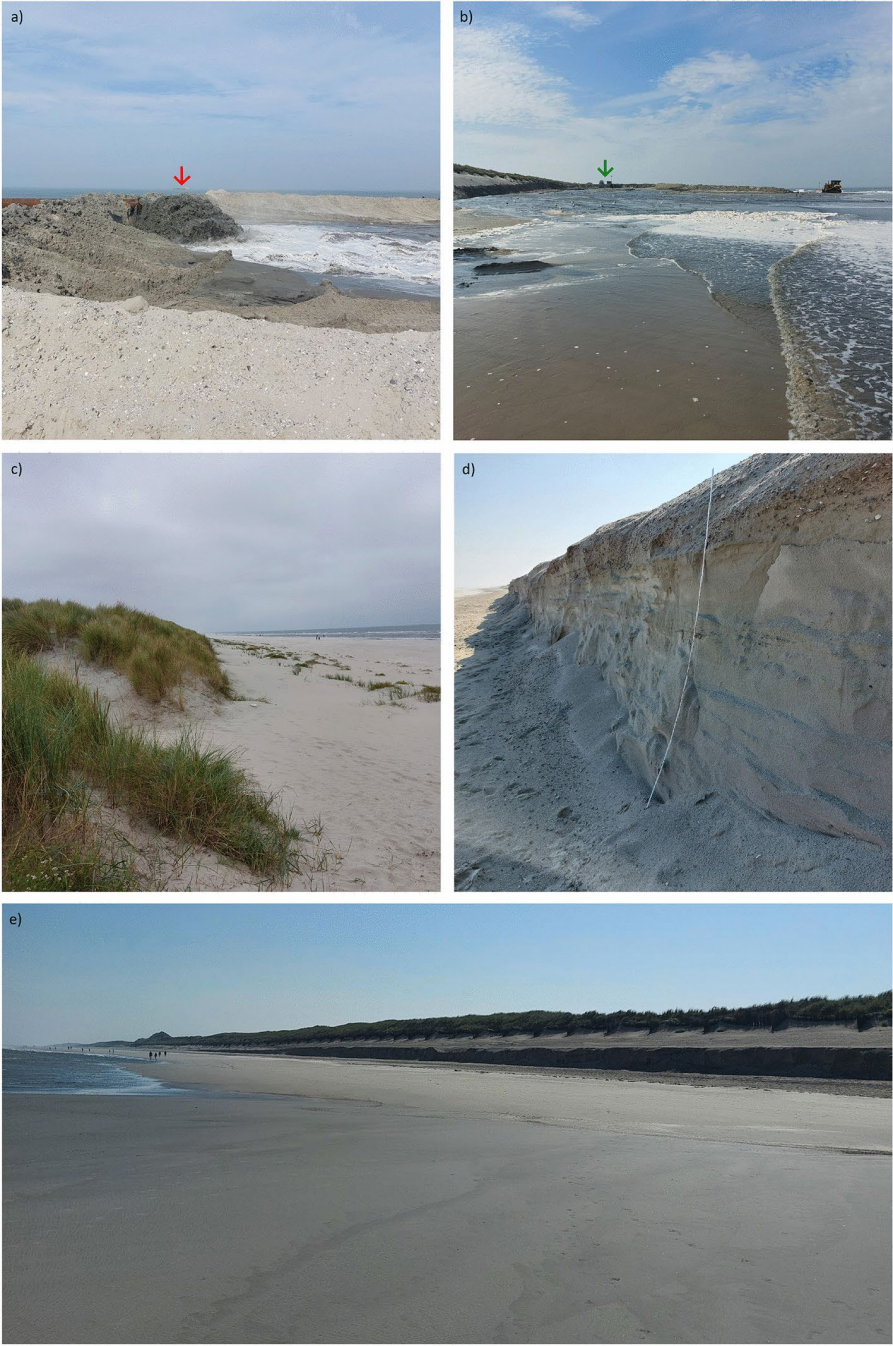
Images of the Island of Langeoog from September 2024. (**a**) Sand replenishment in front of the Pirolatal with the special ship in the background (red arrow) (Image taken on September 7, 2024, 13:48 CET; High Tide: 15:06 CET). (**b**) View of the U-shaped replenishment area facing the flushing pipe (green arrow) (Image taken on September 7, 2024, 14:03 CET; High Tide: 15:06 CET). (**c**) Dune area east of the Pirolatal without significant signs of erosion (Image taken on September 19, 2024, 13:06 CET; High Tide: 13:23 CET). (**d**) Area exposed to severe erosion with a visible dune break-off edge in front of the eastern Pirolatal prior to replenishment (cliff height: 2 m) (Image taken on September 5, 2024, 11:06 CET; High Tide: 14:13 CET). (**e**) Beach section in front of the Pirolatal. The break-off edge of the protective dune, caused by strong local erosion, is clearly visible (Image taken on September 5, 2024, 11:09 CET; High Tide: 14:13 CET). Source: Own photos.

The German Wadden Sea is a natural landscape that is primarily influenced by the tides. The East Frisian Islands located at the seaward edge of the Wadden Sea are of great importance for the German mainland coast. They offer protection from storm surges, support the formation and stability of coastal landscapes, are highly ecologically valuable, contribute to the regional economy and have cultural and historical relevance. Their preservation and protection are therefore of immense importance for the entire region^29^. The islands form a barrier island system in the North Sea, located between 3 and 20 km off the German mainland coast (Fig. 1a); the island system stretches over a distance of 90 km in west-east direction^30^. The East Frisian Islands are characterized by sandy coasts on the seaward side and salt marshes and mudflats on the southern side facing the mainland^31^. As a meso-tidal coastal region, tidal levels must be taken into account when analyzing coastal processes at the Island of Langeoog. The tides in the German Wadden Sea are semi-diurnal. At the tide gauge located at the port entrance of Langeoog, the tidal range is approximately 2.7 m. Mean High Water (MHW) is recorded at 1.38 m, while Mean Low Water (MLW) is -1.28 m (Reference level: *Normalhöhennull (NHN)*). The 99 quantile of the significant wave height in the period 1996 to 2015 is 1.5 - 1.9 m; maximum significant wave height shows strong variations and is up to 4.9 m^32^. Based on the latter value, wave periods show high local variations between 2.5 and 8.5 s. Six main tidal inlets pass between the islands^30^. One of them, the Accumer Ee (Fig. 1b), lies off the west side of Langeoog, one of the seven populated East Frisian Islands^31^. The tidal inlets significantly influence the sediment transport. The predominant sediment type is sand, with median grain sizes between 0.1 and 0.25 mm in the coastal areas and median grain sizes up to 1 mm in the tidal inlets^33^. The main direction of transport points from west to east, where sediments are transported as sand reefs and sand bars. The ebb stream pushes the sand northwards until transport forces of the stream overcome the currents. Then, the transported sediment approaches the northwest beach of the next island in the reef arc^34^. The speed and the amount of sediment transported underlies the influence of several factors, such as tidal range, current, swell and storm surge events. The natural sand supply of the islands depends to a large extent on these transport processes and is therefore also very heterogeneous. Both sand surpluses and sand deficits occur periodicly. The Island of Langeoog is a special case here. The reef arc in front of the western side of the island is very narrow - as a result, the sand bars migrate onshore in the northwest and supply the western beach as well as the northern side of the island (Fig. 1b). Therefore, no protective structures such as groynes have been necessary on the north-western side of Langeoog to date^34,35^. Instead, the island is surrounded by 20.3 km of coastal dunes to the western and northern shores^30^. The dunes differ significantly in their individual shape, depending on prevailing erosion or accretion dynamics (Fig. 2c and d).

In the past, the sand bars migrating onshore were usually sufficient to supply the island’s beaches located in the west and north with enough sand. However, recurring phases of sand shortage and subsequent beach and dune erosion have led to a constant weakening of the protective dune on the north side of the island since the late 1980s^35,36^. This protective dune is crucial for the island’s drinking water supply, as it covers the freshwater lens that supplies the entire island with drinking water^31^. In order to maintain the protective dune and prevent salt water intrusion, regular sand replenishment (Fig. 2a and b) has become necessary in recent years to compensate for the sand deficits. The sand replenishments refill the sand deposit on the sea side in front of the protective dune and raise the beach level. The sand required for this is taken from the east side of the tidal inlet Accumer Ee using a special ship and pumped onto the beach via a flushing pipe. There, the sand is spread with bulldozers. Depending on the severity of the previous storm surge season (October to February), the replenishment activities are carried out in the summer months from June to September of the particular year if necessary. The remaining volume of the sand deposit in front of the protective dune after the winter storm surge season is critical to the necessity of a replenishment measure. The sand deposit is exposed to continuous erosion caused by currents throughout the year. Storm surges occurring in the winter months in particular intensify these erosion processes through increased wave run-up. Annual survey campaigns are carried out in spring to quantify the remaining sand deposit. An overview of storm surges that have occurred in the region and sand replenishment measures on the Island of Langeoog over the past ten years is provided in Table 1.

**Table 1:** Registered storm surges (S) (Deviation from MHW $\ge$ + 1.5 m) at the tide gauge Norderney and sand replenishment measures (R) at Langeoog with corresponding volumes from 2016 to 2024. Usually, replenishment is carried out between June and September. Necessary sand volumes depend on the amount of eroded material during storm surge season in the winter months. Source: Lower Saxony Water Management, Coastal and Nature Protection Agency (NLWKN); Federal Maritime and Hydrographic Agency (BSH).

Year	Replenishment (R) or Storm Surge (S)	Date	Deviation from MHW	Sand volume
2016	S	December 26, 2016 to December 27, 2016	+ 1.91 m	-
2017	S	January 11, 2017 to January 14, 2017	+ 1.66 m	-
2017	S	October 10, 2017	+ 1.65 m	-
2017/2018	R	-	-	200.000 $m^3$
2019	S	January 8, 2019	+ 1.66 m	-
2020	S	February 10, 2020	+ 1.59 m	-
2020	R	-	-	700.000 $m^3$
2022	S	January 29, 2022 to January 30, 2022	+ 1.66 m	-
2022	S	Febuary 17, 2022 to February 22, 2022	+ 2.15 m	-
2022	R	-	-	450.000 $m^3$
2023	S	December 21, 2023 to December 25, 2023	+ 2.19 m	-
2024	R	-	-	450.000 $m^3$

The erosion damage particularly affects the area in front of the Pirolatal (Fig. 2e). Protecting this beach section is crucial for the preservation of the island and the safeguarding of its drinking water supply and settlements. Therefore, it is subject to intensive monitoring by the local coastal protection entity. Surveying and analyses are still carried out using time-consuming in-situ methods, which do not enable continuous monitoring due to their low temporal resolution^37^. Satellite-based remote sensing data with its high temporal resolution would greatly support the survey activities.

## Results

We analyzed HR PlanetScope and MR Landsat-8/9 and Sentinel-2 optical satellite data as well as VHR TerraSAR-X Staring Spotlight SAR data to obtain information on different proxies of coastal dynamics off the sandy shore of the Island of Langeoog regarding the time period 2018 to 2023. We investigate the potential and limitations of different sensor types and methods to gain findings regarding several proxies of coastal dynamics, like onshore migration of sand bars, changes of the instantaneous waterline and sandy beach areas as well as the position of the landward dune toe. The data and methods are described in detail in the *Material and Methods* section.

### Identification and change analysis of sand bars

**Fig. 3:**
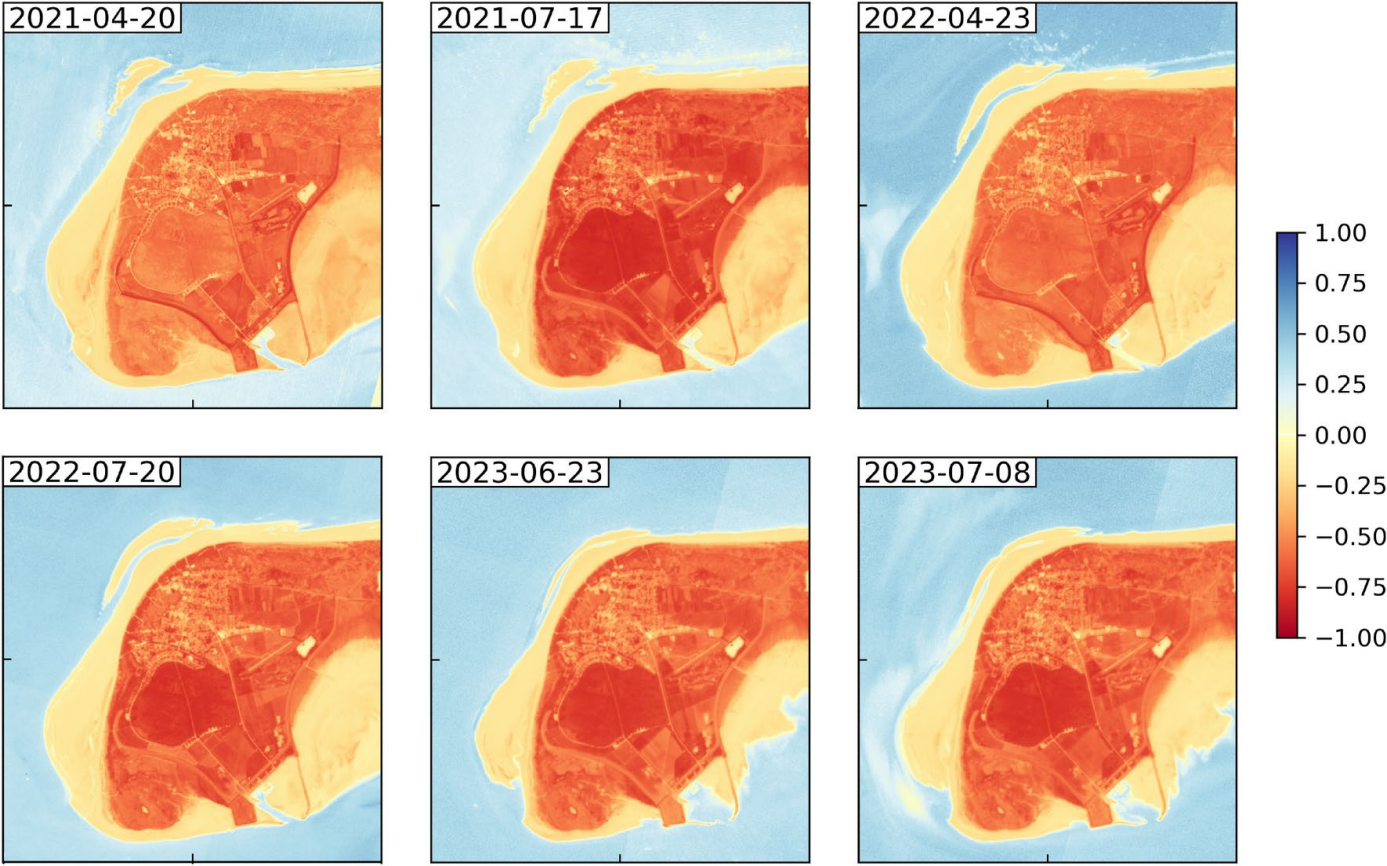
NDWI for six HR PlanetScope acquisitions in the period April 20, 2021 to July 8, 2023, showing onshore migration of a sand bar at the northwestern tip of the Island of Langeoog. Changes along the instantaneous waterline of the sandy beach areas are also visible as well as variations at the southern shores of the island. Corresponding tide levels are displayed in Table 2. Processed satellite imagery: PlanetScope (Planet Labs 2021-2023). Map created using Matplotlib 3.9 (https://matplotlib.org/).

The main accretion processes occur as sand bars migrating onshore at the north-western tip of the Island of Langeoog due to the shape of the reef arc. We have therefore focused on the north-western tip of the island for the analysis of this proxy. For HR data, we performed a threshold technique based on the NDWI (Normalized Difference Water Index) (“*Analysis of the instantaneous waterline, dune toe detection and estimation of dry sand areas from HR optical satellite imagery*” section for details). For MR data, we expanded analysis to two further indices suitable for water detection due to the wider radiometric resolution of MR sensors: MNDWI (Modified Normalized Difference Water Index) and AWEI (Automated Water Extraction Index) (“*Analysis of the instantaneous waterline and dune toe detection from MR optical satellite imagery*” section for details). The indices are derived using different spectral bands and can be interpreted in a similar way: Water surfaces assign values > 0, land surfaces values < 0. Applying each of the indices, we were able to extract sand bars located off the northwestern coast of Langeoog from the data and trace the movement of a sand bar that approached Langeoog’s coast in the period 2020 to 2023 and merged with the beach in 2023 (Fig. 3; Table 2). From April 20th, 2021 onwards, HR data shows the continuous migration of the sand bar towards the coast at a rate of 15 m ± 3.7 m per month. During this process, its shape changes: the length of the sand bar (northwest-southeast) steadily decreases, while its width increases. Driven by the prevailing current conditions, the sand bar is transported from the reef arc towards the northwest side of the island, where it merges with the beach. The image from July 8, 2023, shows an existing connection between the sand bar and the coast at low tide. At higher water levels, however, it may still be separated from the island by the visible tidal inlet.

**Table 2:** Tide levels for the six PlanetScope acquisitions plotted in Fig. 3. Acquisition time in CET, tide level measurements are referenced to *Normalhöhennull* (NHN). Low tide values present the water level at the closest preceding or upcoming low tide relative to acquisition time. Source: Federal Waterway and Shipping Administration (WSV), provided by Federal Institute of Hydrology (BfG).

Aquistion Date	Acquisition Time	Tide Level during Acquisition	Low Tide
2021-04-20	11:09	$-$0.90 m	$-$1.18 m
2021-07-17	11:37	$-$1.09 m	$-$1.33 m
2022-04-23	11:27	$-$1.23 m	$-$1.45 m
2022-07-20	11:19	$-$1.12 m	$-$1.31 m
2023-06-23	11:15	$-$0.06 m	$-$1.25 m
2023-07-08	11:32	$-$0.09 m	$-$1.49 m

We also investigated the differences between the indices. Results are shown in Table 3. When comparing the three indices with each other, standard deviations show in general low values between 0.073 for MNDWI compared to AWEInsh results and 0.112 for NDWI compared to AWEInsh results. Differences occur in the intermediate area of the land-water interface. With the NDWI, most pixels (55.09%) are classified as land, with the AWEInsh a higher percentage are categorized as water (56.57%). MNDWI shows values lying in between for land and water pixel distribution. The NDWI is best at mapping sand bars off the coast: as with MNDWI and AWEInsh more pixels are categorized as water, very small sand bars may not be visible in the results. Furthermore, MDNWI and AWEInsh can only be calculated for MR data. The coarser spatial resolution of these data also leads to reduced visibility of sand bars. The AWEInsh is more accurate at identifying tidal channels due to higher proportion of water pixels detected. With the NDWI, tidal channels may not be visible due to the overestimation of land pixels in the intermediate area of the land-water interface.

**Table 3:** Standard deviations for NDWI, MNDWI and AWEInsh calculated for a Sentinel-2 scene (Acquisition date: June 11, 2023) and distribution of pixels identified as water and land area per index [%].

NDWI - MNDWI	NDWI - AWEInsh	MNDWI- AWEInsh
0.097	0.112	0.073

	land pixel count [%]	water pixel count [%]
NDWI	55.09 %	44.91 %
MNDWI	47.26 %	52.74 %
AWEInsh	43.13 %	56.87 %

**Fig. 4:**
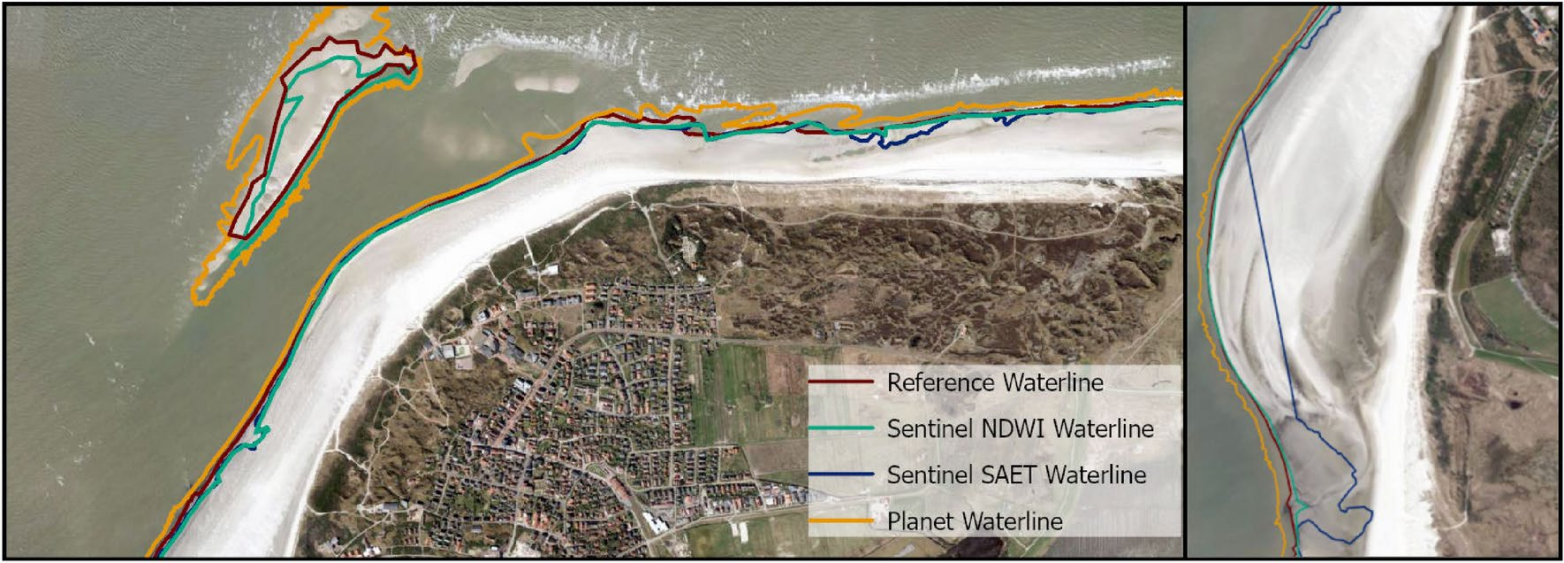
Sand bar and instantaneous waterline extracted from different data sources for May 30, 2020. Corresponding tide levels are given in Table 4. Basemap: DOP20 (acquisitioned on April 19, 2020) (Source: GeoBasis-DE/LGLN2024). Map created using ArcGIS Pro 3.2.0 (https://www.esri.com/).

For a dataset acquired on May 30, 2020, we compared the results of extracting a sand bar from HR and MR optical data (Fig. 4). The extracted sand bars were referenced against a reference sand bar derived from a digital orthophoto (DOP) acquired on April 19, 2020. Since the datasets were acquired at different time points, and the DOP was captured on a different date, differences in tide levels must be considered when interpreting the results (Table 4). All three extracted sand bars intersect with one another. The calculated centroids of the extracted sand bars lie 22.26 m (Reference - HR), 42.10 m (Reference - MR), and 36.76 m (HR - MR) apart from each other. The largest measured area corresponds to the sand bar extracted from the HR data, while the MR-extracted sand bar and the reference sand bar exhibit smaller area values. Variations in the extracted areas may be attributed to differences in tide levels during data acquisition (Table 4). The water level at the time of HR data acquisition was significantly lower than that for the other two datasets. Since the reference sand bar and the sand bar extracted from MR data display comparable area values, the southeastward offset of the sand bar extracted from HR data may indicate the onshore migration observed in the time series.

**Table 4:** Tide levels for results plotted in Fig. 4. Acquisition time in CET, tide level measurements are referenced to *Normalhöhennull* (NHN). Low tide values present the water level at the closest preceding or upcoming low tide relative to acquisition time. *Due to tide gauge failure, there are no measured values between March 19, 2020 and April 21, 2020; low tide estimation from tidal forecast of Federal Institute of Hydrology (BfG). Source: Federal Waterway and Shipping Administration (WSV), provided by Federal Institute of Hydrology (BfG).

Data Source	Aquistion Date	Acquisition Time	Tide Level	Low Tide
DOP20	2020-04-19	-*	-*	$-$1.13 m*
Planet (HR)	2020-05-30	10:47	$-$1.33 m	$-$1.39 m
Sentinel-2 (MR)	2020-05-30	11:50	$-$1.23 m	$-$1.39 m

### Identification and change analysis of the instantaneous waterline

**Fig. 5:**
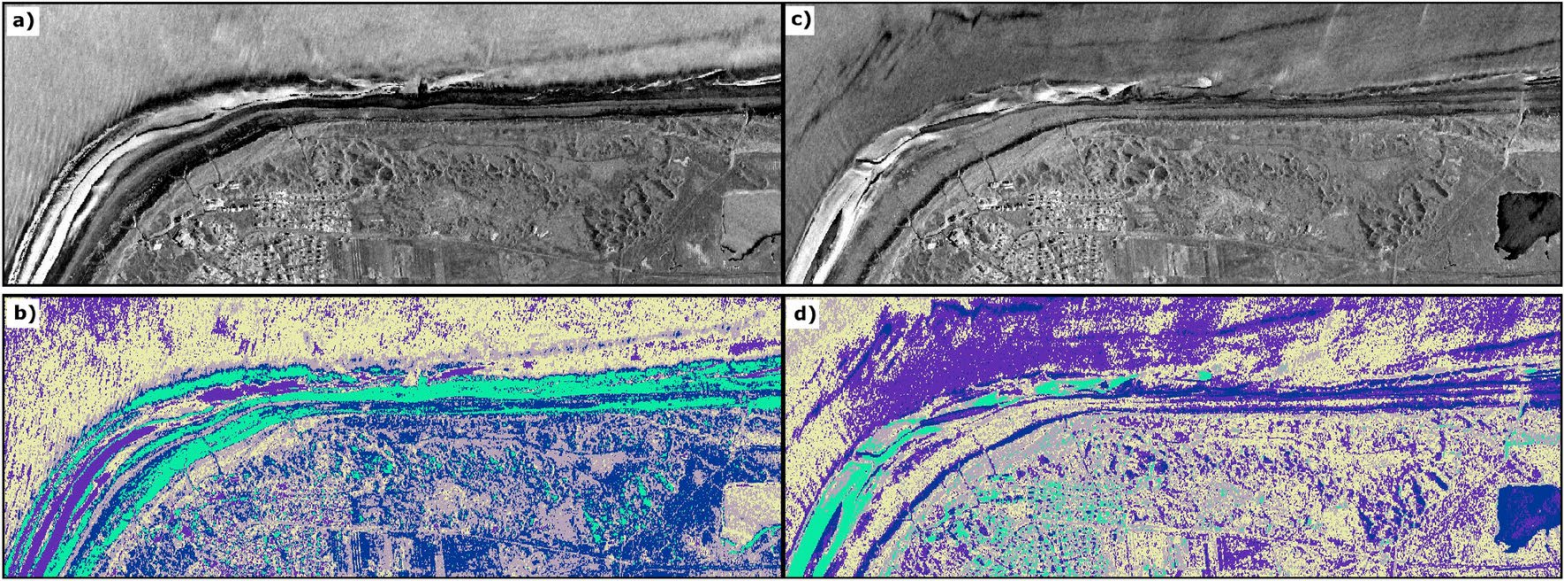
(**a**) TerraSAR-X Staring Spotlight acquisition from May 19, 2023 (Acquisition time: 07:00 CET, tide level = 4.00 m, low tide = 3.37 m) (**b**) Unsupervised classification of TerraSAR-X Staring Spotlight acquisition from May 19, 2023. (**c**) TerraSAR-X Staring Spotlight acquisition from August 15, 2023 (Acquisition time: 07:00 CET, tide level 4.73 m, low tide = 3.87 m) (**d**) Unsupervised classification with 5 classes of TerraSAR-X Staring Spotlight acquisition from August 15, 2023. Results indicate significant perturbations in the HH-polarized data at the beach-water interface, caused by variations in surface roughness due to wave action. These effects are further amplified by the alternating presence of tidal channels and sandbars. The beach-inland transition is visually discernible in the results. Processed satellite imagery: TerraSAR-X/TanDEM-X DLR e.V. (2023). Map created using ArcGIS Pro 3.2.0 (https://www.esri.com/).

**Fig. 6:**
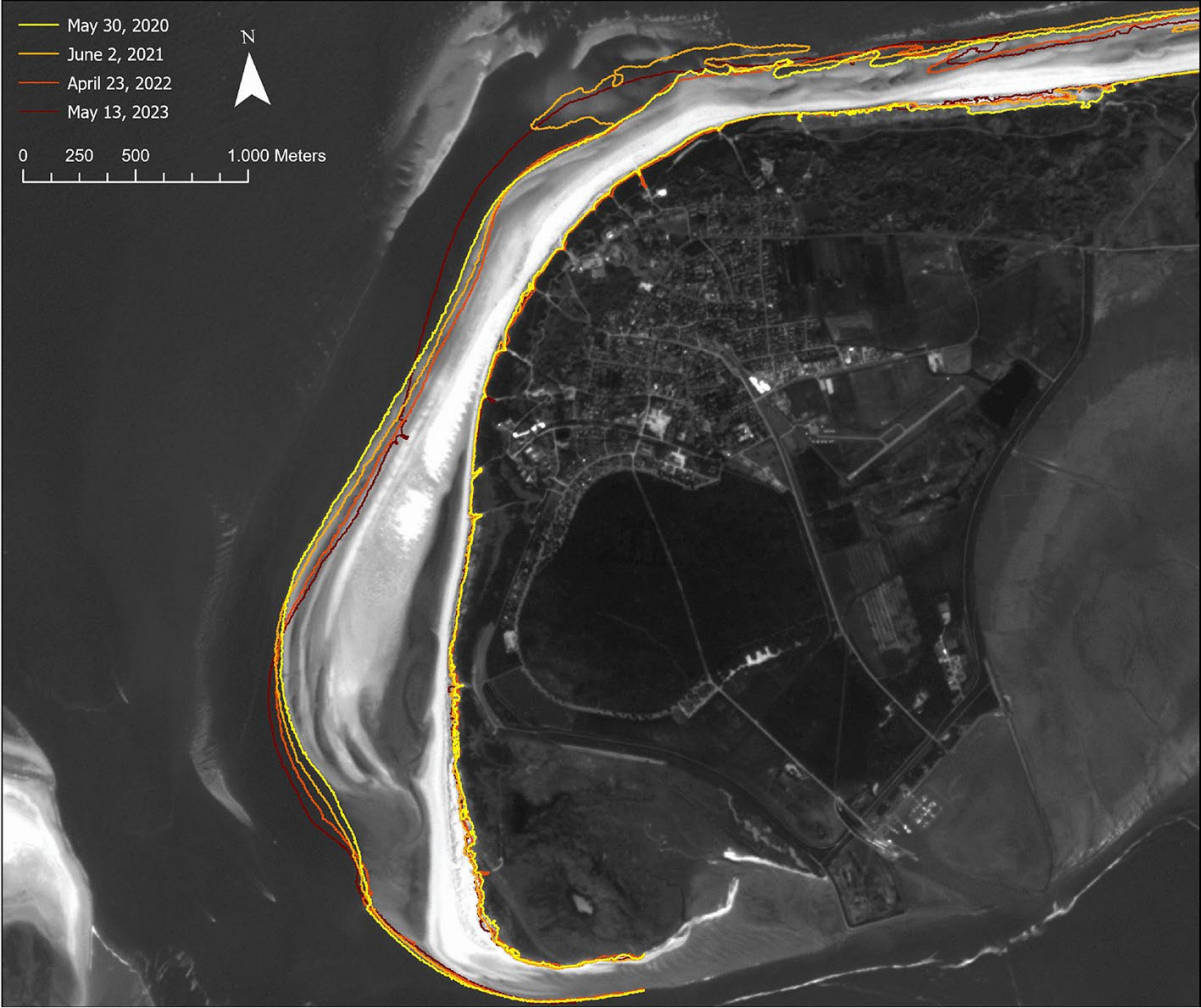
Instantaneous waterline and dune toe position derived from four PlanetScope acquisitions in the time period 2020 to 2023. Background shows the PlanetScope acquisition from May 30, 2020. Corresponding tide levels are displayed in Table 5. Processed satellite imagery: PlanetScope (Planet Labs 2020). Map created using ArcGIS Pro 3.2.0 (https://www.esri.com/).

For investigating changes of the instantaneous waterline and subsequent changes of the shoreline at the Island of Langeoog, we used our previous results to extract waterlines from HR and MR optical data (“*Analysis of the instantaneous waterline, dune toe detection and estimation of dry sand areas from HR optical satellite imagery*” and “*Analysis of the instantaneous waterline and dune toe detection from MR optical satellite imagery*” sections for details). Additionally, we used the tool SAET (Shoreline Analysis and Extraction Tool)^38^ to investigate a fully automated method for processing MR optical data to obtain shoreline information. We also attempted to incorporate VHR SAR data into the analysis, as its high spatial resolution holds the potential to yield more accurate results for waterline extraction. However, initial tests on classifying the SAR data revealed limitations of HH-polarized data in combination with the chosen method for our use case (Fig. 5). Strong perturbations at the land-water interface hinder the extraction of meaningful information related to the waterline. These initial findings highlight the fundamental challenges associated with shoreline extraction in this environment. By analyzing optical data, we observed a westward shift of the extracted instantaneous waterlines at the west side of the island, during the full time period of 2018 to 2023. These results indicate a permanent westward shift of the shoreline due to the growth of the south western sand depot (Fig. 6). Changes at the northwestern tip of the island and the northern coast show stronger fluctuations, as they are strongly influenced by alternating accretion and erosion events. The irregular coastal shape in the beach section, characterized by small sand bars lying offshore and tidal channels inbetween which get drained and flooded periodically depending on tide level, complicates the identification of a clear trend. We observed no significant impacts of sand replenishment actions taking place at the coast before Pirolatal to the shoreline, as the working area is still identified as sand area. It must be noted in that context, that there may be some visible influence of the measure to the instantaneous waterline during high tides, which we did not asses within this work. Comparisons between the different sensor types and methods highlight the influence of tidal levels, which must be carefully considered when interpreting the results. Mean distance between HR derived waterline and MR NDWI waterline for acquisitions on May 30, 2020 (Fig. 4) is 18.42 m (standard deviation 16.43 m) with a water level difference of + 0.10 m for MR data to be considered. Visual results confirm that difference as MR derived waterline shows a landward offset to HR derived waterline. Mean distance between HR and MR SAET derived waterline shows higher values (mean distance 52.16 m, standard deviation 77.13 m) due to uncertainties at the western beach section (Fig. 4). Comparison of the results with reference data shows mean deviations of 10.14 m for MR derived NDWI waterline up to 22.76 m for HR derived waterline (Table 6). Variations underlie substantial short-term influence of tidal water level changes.

**Table 5:** Tide levels for the PlanetScope acquisitions plotted in Fig. 6. Acquisition time in CET, tide level measurements are referenced to *Normalhöhennull* (NHN). Low tide values present the water level at the closest preceding or upcoming low tide relative to acquisition time. Source: Federal Waterway and Shipping Administration (WSV), provided by Federal Institute of Hydrology (BfG).

Aquistion Date	Acquisition Time	Tide Level during Acquisition	Low Tide
2020-05-30	10:47	$-$-1.37 m	$-$1.39 m
2021-06-02	10:59	$-$1.35 m	$-$1.40 m
2022-04-23	11:27	$-$1.23 m	$-$1.45 m
2023-05-13	11:19	$-$1.23 m	$-$1.31 m

**Table 6:** Deviations of the extracted instantaneous waterlines from the reference line (derived from DOP20).

Data	Mean	Standard Deviation
Sentinel NDWI Shoreline (MR)	10.14 m	16.43 m
Sentinel SAET Shoreline (MR)	33.47 m	54.37 m
Planet Shoreline (HR)	22.76 m	12.54 m

### Identification and change analysis of the dune toe

**Fig. 7:**
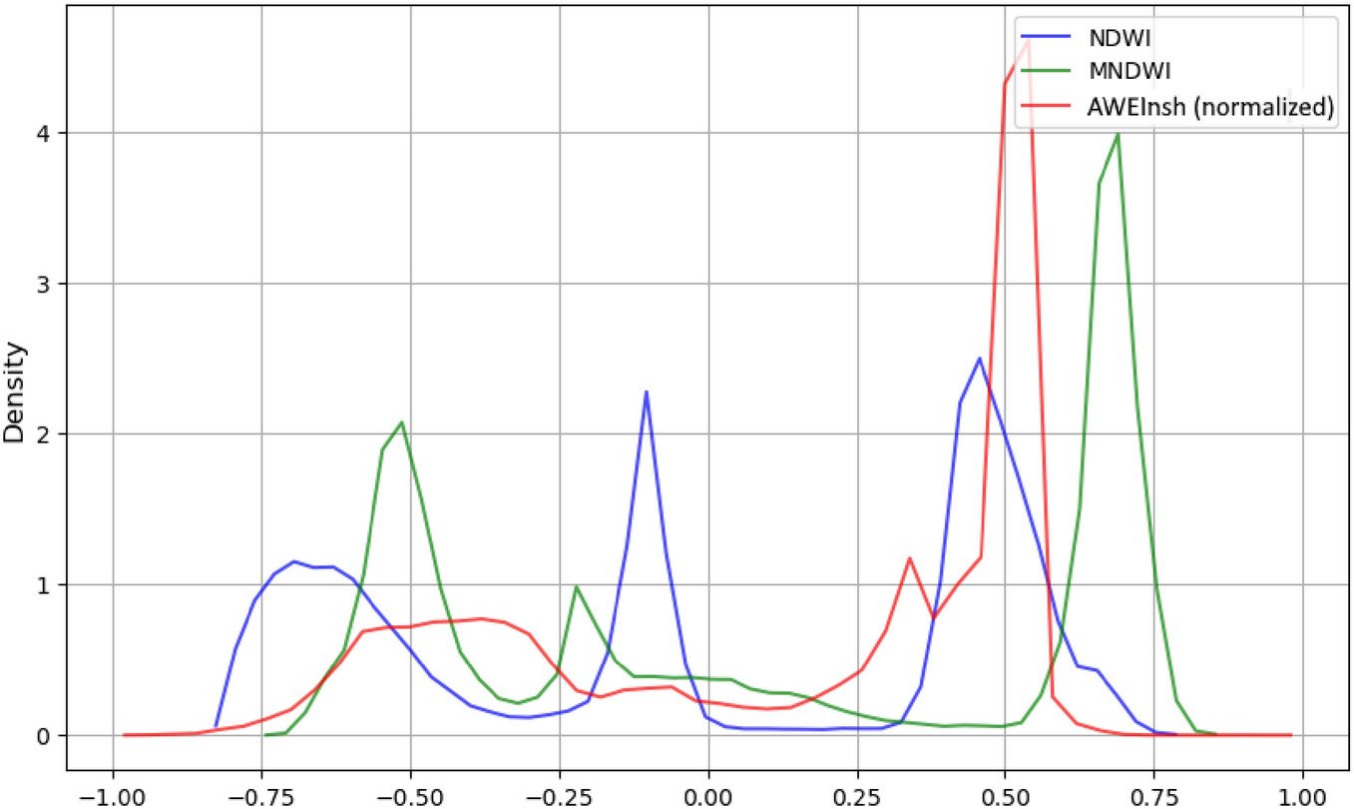
Density plots for three water indices calculated from a Sentinel-2 acquisition taken on June 11, 2023. AWEInsh is normalized for better comparison.

For the detection and change analysis of the dune toe, we analyze HR and MR optical data as well as VHR SAR data using thresholding methods (“* Analysis of the instantaneous waterline, dune toe detection and estimation of dry sand areas from HR optical satellite imagery*”, “*Analysis of the instantaneous waterline and dune toe detection from MR optical satellite imagery*” and “*Dune toe extraction from VHR SAR data*” sections for details). We investigate the time period 2022 to 2023 here in particular, as there were several storm surges in the beginning of year 2022 as well as a sand replenishment measure starting in June 2022. Regarding the optical data, we examine the calculated indices for their suitability for detailed thresholding methods. Densities (Fig. 7) show that the NDWI is particularly suitable for multi-modal threshold analysis: The negative values of the index for land areas can be further subdivided into sand areas and other land cover due to the different spectral properties of the surfaces. The resulting boundary between the two value ranges is assumed to be the dune toe (Fig. 6; Table 5).

Results for optical data show differing patterns for extracting the dune toe at the western and northern beach areas. In the southwestern section, fluctuations in the results indicate changes over the year regarding the dune toe. These patterns may be explained by changing land cover (e.g., vegetation growth) which leads to differing spectral information and therefore a change in NDWI values. In the northwestern section, no distinct changes are visible. In front of Pirolatal, results show a seaward shift of the dune between 2020 and 2023. This shift may also be related to a change in the dune toe position, but changes in land cover are also possible. Additional topographic or DEM data may be useful for future investigations. In both cases, we have identified significant environmental changes in the dune section in front of Pirolatal. No significant changes in the dune toe that could be linked to erosion or accretion events were detected either between the years or in the detailed analysis for 2023.

**Fig. 8:**
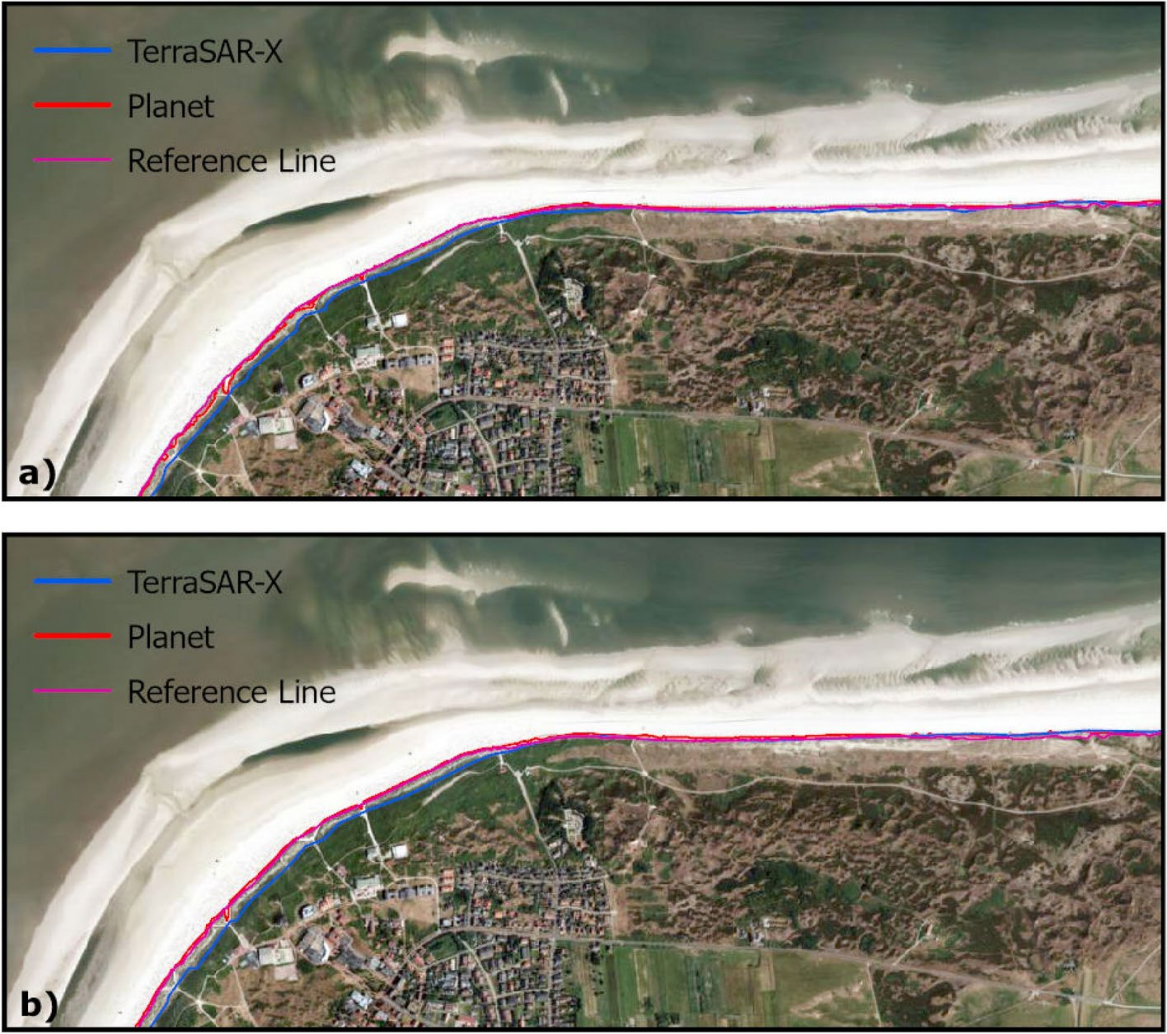
Results for dune toe extraction from VHR SAR and HR optical data. (**a**) Results for data takes from May, 2023 (TerraSAR-X: May 19, 2023, Planet: May 13, 2023) (**b**) Results for data takes from August, 2023 (TerraSAR-X: August 15, 2023, Planet: August 11, 2023) Basemap: DOP20 (acquisitioned on July 8, 2023) (Source: GeoBasis-DE/LGLN2024). Map created using ArcGIS Pro 3.2.0 (https://www.esri.com/).

For the year 2023, an analysis was conducted to assess potential interannual shifts in the position of the dune toe. The investigation utilized VHR SAR-data supplemented by optical data. Results indicate that the dune toe position remained stable throughout the year. To validate these findings, two samples of dune toe positions extracted from VHR SAR data were compared with the corresponding dune toe information derived from optical data using NDWI information (Fig. 8). The analysis was referenced against a baseline dune toe position, which was extracted from a DOP. The mean distance between the reference dune toe and the dune toe extracted from optical data was 463 m for the acquisition in May (689 m for the acquisition in August), with a standard deviation of 367 m (296 m). The minimum recorded distance was 0.14 m (087 m), while the maximum was 16.61 m (1631 m). The distances remained consistent across the study area, with larger deviations observed primarily at beach access paths.

The mean distance between the reference data and the values extracted from the VHR SAR data is found to be higher: 1579 m (1507 m), with a standard deviation of 12.22 m (1417 m). The minimum distance is 066 m (000 m), and the maximum distance is 4003 m (5956 m). Regarding the extracted dune toes, distinct patterns are observed between the northern dune toe section and the western path, with the differences in the western path being significantly larger. This discrepancy can be attributed to the varying topographic characteristics of Langeoog’s protective dune. The steep break-off in the northern section allows for high precision extraction, while the shallow slopes in the northwest and west, characterized by heterogeneous surfaces, contribute to errors in estimating the dune toe.

### Estimation of permanently dry sandy beach areas

**Fig. 9:**
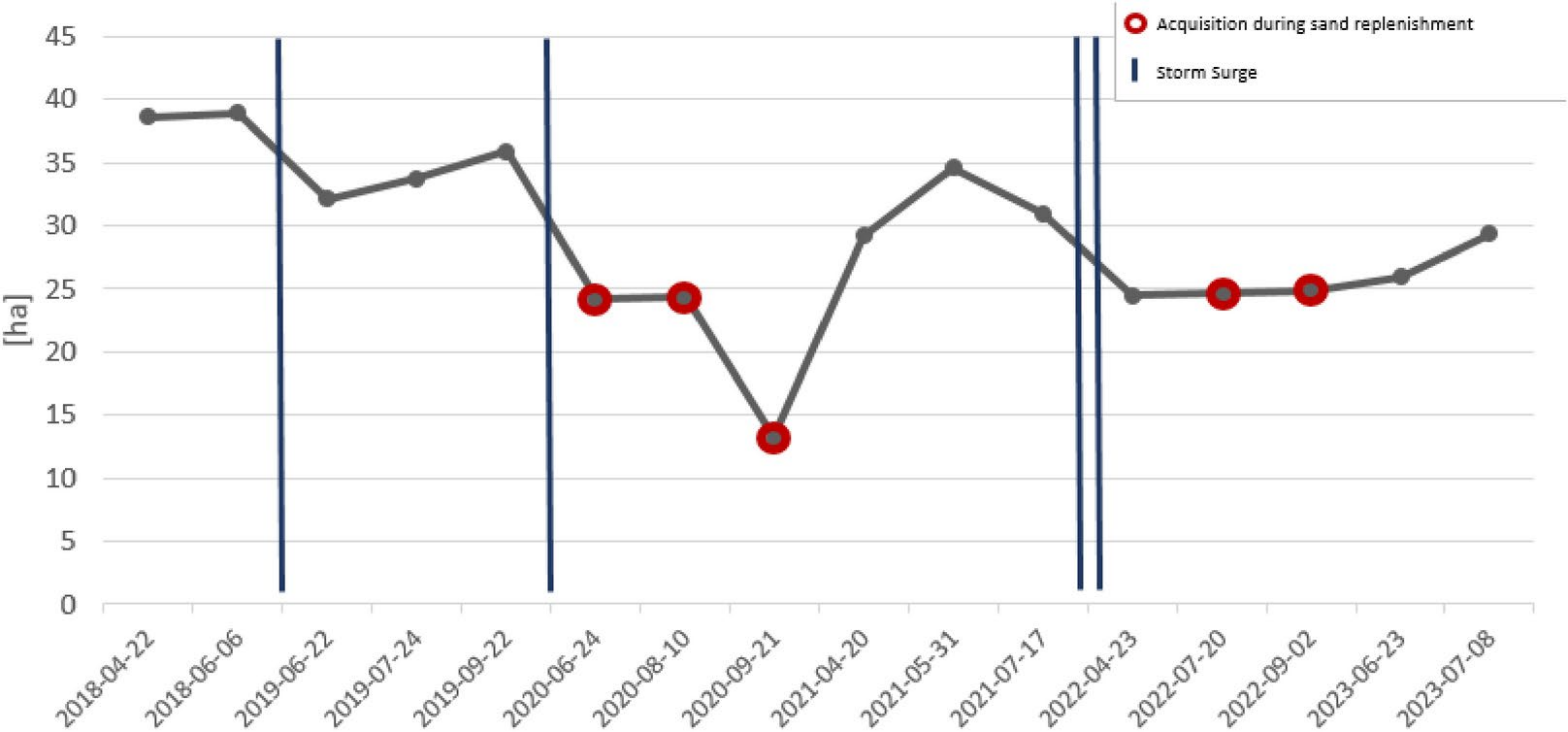
Dry sandy beach areas off the Pirolatal in the period April 2018 to July 2023. Acquisitions marked in red were taken during sand replenishment. Blue lines mark storm surge events that took place between acquisitions.

For estimating permanently dry sandy beach areas, we used results of the class “*Dry Sand*” extracted by a Random Forest classification workflow. We focused on the beach area in front of Pirolatal, as it is the beach section mostly affected by erosion and therefore the area where sand replenishments take place. Our results (Fig. 9) in general indicate impacts of sand replenishments: The execution of the measure may lead to a significant reduction of dry sand areas in front of Pirolatal during the execution of the replenishment, followed by an increase of dry sand areas after finishing the measure. There is also a visible influence of storm surges during winter season, eroding the sand depot before Pirolatal. For the replenishment measure in 2020, a significant reduction in dry sand areas can be observed in the preliminary phase during the storm surge season. This reduction is further enhanced by the implementation of the replenishment (Fig. 9, September 21, 2020). Once the measure has been completed, the dry sand areas are restored and their significant increase is still visible in the following year. Results differ for the replenishment in 2022. While a significant reduction in dry sand areas is also visible here during the storm surge season, areas remain stable during the execution of the measure. This behavior may be related to the temporal resolution of the study, as the replenishment does not always take place in similar time frames and sequences from year to year and there may have been further reduction in dry sand areas between the data acquisition dates. After completion of the measure, an increase of dry sand areas can also be observed here in the following year 2023.

## Discussion

There are numerous types of sandy coastal environments, each with specific characteristics regarding e.g. topography, tidal properties and wave conditions^1^. The East Frisian Islands represent a meso-tidal coastal environment characterized by a highly dynamic interaction of tidal range, marine current patterns, and seasonal storm surge activity^30^. Applying global models in such environments proves challenging, as these methods may not adequately capture the individual components of coastal dynamics^39^. In a meso-tidal coastal environment, short-term changes in coastline proxies due to fluctuating tides or varying wave conditions pose significant challenges when assessing coastal features like the shoreline or beach conditions. Additionally, restricted availability of optical data, often caused by poor weather conditions and frequent cloud cover typical of coastal areas, complicates efforts to achieve high temporal resolution in change analysis. Furthermore, data availability is often unevenly distributed throughout the year^39^, as cloud cover tends to be more prevalent during winter months. This seasonal limitation reduces the potential to assess the immediate impacts of storm surges, which typically occur during the winter in our study area. Both limitations are well recognized and acknowledged as some of the most significant challenges in remote sensing-based coastal monitoring within the scientific community. Utilizing multiple satellite remote sensing data sources offers the potential to simultaneously leverage the advantages of various sensor types while mitigating the limitations associated with relying on a single data source. The advantage of HR optical satellite data lies in its high spatial resolution, which enables the assessment of small-scale patterns along the waterline and the identification of small emerging sand bars. However, limitations include restricted accessibility due to predominantly commercial missions and, as with optical data in general, the requirement for daylight and favorable weather conditions. MR optical data offers advantages in terms of data accessibility and extensive archives that provide long time series suitable for coastal monitoring. Its high radiometric resolution supports the application of various methodologies and the integration of multiple indices. Additionally, the large volume of available data makes it well-suited for processing in automated environments. The primary limitation of MR data is its relatively coarse resolution, which constrains its utility for analyzing small-scale patterns. Overall, HR data enhances temporal resolution by condensing time series, providing more detailed insights. It is widely used to analyze coastal dynamics across various coastal environments, including micro-, meso-, and macro-tidal beaches^13,39^. Since SAR sensors collect data independently of weather conditions and daylight^22^, the VHR TerraSAR-X Staring Spotlight dataset enabled us to produce a dense time series for 2023. The suitability of different polarization options combined with different methods in SAR data is highly application-specific. For instance, HH-polarized SAR data was not applicable for assessing all proxies demonstrated in this study. SAR data is widely utilized for analyzing coastal dynamics. Several studies use VV-polarized data for waterline extraction^4^, while others have experimented with various dual-polarization modes^23^. Leveraging SAR data, particularly the VHR capabilities of TerraSAR-X’s Staring Spotlight mode, presents further potential for advancing assessment of coastal dynamics by also testing further methodological approaches. The primary challenge in this study is extracting and interpreting meaningful information in the absence of in-situ measurements for validation. Local authorities conduct an assessment of the current beach state once a year (typically in late spring or early summer) to estimate storm surge damages from the preceding winter season. However, these measurements are not publicly accessible. To ensure the comparability and reliability of results in such regions, employing multiple sensor types and diverse methods allows for cross-validation of the findings. The lack of continuous in-situ data underscores the critical value of remote sensing data in such use cases.

To extract the instantaneous waterline and identify migrating sand bars, we employed an index-based thresholding approach, testing multiple indices to optimize results. Accurately determining the waterline through its various proxies and relating it to the position of the actual shoreline is a central focus in remote sensing research on coastal dynamics. In this study, we followed established methodologies to identify proxies that approximate the actual shoreline’s location. Baiocchi et al.^15^, for example, highlight several proxies, including the wave-breaking line, the instantaneous waterline (commonly regarded as the most widely used proxy), and the boundary between dry and wet sand. Extracting instantaneous waterlines and linking them to long-term shoreline trends presents a significant challenge in our study area due to the lack of in-situ reference data for validation. However, this also makes the area representative of many coastal regions worldwide, where in-situ measurement data are similarly insufficient. To address this limitation, we focused on analyzing data during low tide to ensure comparability of our results. At the same time, the images captured during low tide allow for the estimation of sandbars offshore that are not visible during high tide. Nonetheless, varying tidal levels must be taken into account when interpreting the findings of this study. Luijendijk et al. (2018) analyzed global shoreline changes and presented their results in a freely accessable web application^1^. They processed MR optical satellite imagery in a time series since 1984 by applying a pixel-based supervised classification method. The findings of that study for the Island of Langeoog support our results: The study identifies the beach section in front of Pirolatal as threatened by ongoing erosion. The erosion rates lie between -3.4m/yr +- 1.2 m and 6.5 m/yr +- 0.8 m. High erosion rates are also indicated within the tidal channel of Flinthörn (up to 7.5 m/yr +-1.7 m). Here, however, the reference line runs within the western sand bank, which continues to grow according to our results. Thus, regarding results from Luijendijk et al.(2018), only the tidal channel recedes inland, but the actual shoreline moves seaward. This accretion trend can be observed further north on the west coast, as accretion rates of 8.6 m/yr +- 3.0 m are indicated here. As in our approach, no statements can be made by Luijendijk et al. (2018) about the volumes of eroded or accreted material. Three-dimensional analyses require the availability of (tri-)stereo optical images for processing digital elevation models or bi-static InSAR data. The susceptibility to errors in extracting the waterline may be associated with shallow water areas and low beach slopes, as also noted by Haagenaars et al.^40^ Additionally, run-up dynamics, tidal channels, and sand bars contribute to uncertainties in waterline extraction. Konstantinou et al. (2023)^39^ found that applying appropriate water level corrections significantly improved the accuracy of satellite-derived shorelines. Specifically, for the dissipative study site (which shares characteristics with Langeoog’s sandy beach coast), they found that accounting for wave-induced water level fluctuations, including wave setup and run-up, was more crucial than considering tidal elevation. For the results obtained using the SAET tool, another limitation arose due to the specific morphological characteristics of our study area: The SAET tool^38^ we used for extracting the instantaneous waterline from MR optical data benefits from its high degree of automation and overall provides comparable results, but suffers from dependency on the European Coastal Zone Dataset, which leads to weaker results at coastal sections that are not marked as sandy beaches in this dataset ( i.e. the western sandbank on the Island of Langeoog). The analysis of nearshore sand bars migrating onshore in our study focused on delineating the land-water boundary of these sediment bodies to estimate their two-dimensional size, evolution, and movement rate. For volumetric estimations, additional analysis using bathymetric data is required. Alevizos (2024)^41^ introduces a novel method for processing pseudo-bathymetric data from optical drone imagery, enabling the generation of bathymetric information in regions lacking high-resolution measurements. Testing the transferability of this approach to satellite remote sensing data could help evaluate the potential of deriving temporally high-resolution bathymetric data from optical satellite imagery for application in study sites where bathymetric data is unavailable.

The analysis of the foredune and dune toe was conducted using two-dimensional optical and SAR data. Uncertainties arise due to variations in the topographic characteristics of the foredune, with higher uncertainties observed in areas with flat dune slopes compared to sections with steep break-offs caused by severe erosion. As the transition point between the beach and the dune, the dune toe provides critical information for assessing dune dynamics. Elevation data plays a key role in accurately identifying the dune toe and is commonly used to evaluate the state and changes in beach-dune systems^42,43^. Examinging methods to compensate for the missing elevation data has the potential to enhance findings when analyzing dune toe patterns.

The analysis of the dry sandy beach area was based solely on data derived from HR imagery. This represents a significant limitation of the study, as no in-situ measurements were available for validation purposes. Consequently, the accuracy of the results remains uncertain. The approach presented here constitutes a first attempt to quantify dry sandy beach areas using remote sensing data, and as such, the associated uncertainties must be acknowledged and taken into consideration. Despite these limitations, the results demonstrated logical connections to known patterns, such as the impacts of storm surges and beach replenishment measures. This alignment suggests that the derived data could serve as a valuable basis for an initial assessment of the efficiency of such measures. In particular, this information could support local coastal protection authorities in their decision-making processes. Nevertheless, further investigations and validation efforts are essential to enhance the reliability and applicability of the results. Several potential improvements have been identified. First, limitations during the winter months, which coincide with the storm surge season, must be addressed. During this period, high cloud cover frequently impedes the availability of optical remote sensing data. Currently, erosion processes can only be identified through differences observed between fall and spring datasets. Improving data availability during winter months would enable a more continuous monitoring of changes in the dry sandy beach area. Second, the visibility of the influence of replenishment measures is highly dependent on the timing of image acquisition and the temporal resolution of the available data. Since the execution of replenishment measures occurs over a period of time, higher temporal data density is required to accurately capture and assess these changes. Increasing the frequency of data acquisition would thus enhance the ability to track the progress and impact of such measures. Another potential improvement involves the integration of elevation data. Changes from the “*Dry Sand*” beach class to the “*Wet Sand*” class can be driven by two distinct processes: (1) a lowering of the beach surface level, for example, due to erosion from storm surges, and (2) an artificial increase in water levels over an extended period, as observed during replenishment activities. Incorporating elevation data could support the differentiation between these two processes and serve as a validation tool to improve classification accuracy. Finally, the inclusion of radar data is proposed as a means to improve analysis capabilities. Specifically, testing additional polarizations, such as VV or dual- polarization (dual-pol), could provide further insights into the characterization of dry sandy beach areas. The integration of SAR data, with its ability to penetrate cloud cover, would also help to overcome the aforementioned limitations associated with optical data availability during winter months. This approach warrants further exploration to assess its full potential for monitoring and quantifying changes in dry sandy beach areas.

## Conclusions

Detailed information about coastal dynamics is crucial for the monitoring of coastal environments and therefore for the application of effective coastal protection measures. In this study, we investigated the suitability of multi-sensor remote sensing data for monitoring coastal dynamics in highly dynamic meso-tidal sandy coastal environments. For that purpose, we analyzed several proxies to estimate accretion and erosion patterns at the Island of Langeeog. As a key accretion process, we identified the onshore migration of sand bars at the north-western tip of the island. We analyzed the speed of movement and also tracked movement caused changes in the shape of the sediment body. The merging of the sand bar with the island’s beach could be linked to a seaward change of the instantaneous waterline. Our results indicate a long-term influence of sand bar migration and the underlying currents on the position of the shoreline. The strong impact of short term changes in tides and waves must always be considered when interpreting these results. Furthermore, we identified changes in dry sandy beach areas, which can be linked to extreme weather events in the winter months, leading to a reduction of these areas, and replenishment measures in summer time, restocking the sand depots. These results may also be linked to position changes of the shoreline. But with poor data availability in the winter months, the immediate impact of storm surges is not clearly detectable. Results for the influence of replenishment measures indicate an impact of the shoreline position, but need further analysis of additional elevation data to be confirmed. For the position of the dune toe, we found no significant change patterns during the research period. There may be future potential analyzing additional three-dimensional data, as at present, only analysis of the horizontal shift of the dune’s break-off is feasable. In total, our results confirm the landwards decreasing impact of tides and waves on the coastal zone. Whereas strong impacts can be observed on migrating sand bars and the instantaneous waterline, influence on dry sandy beach areas is limited to events beyond average tidal conditions. There is no measurable impact on the state of the dune apart from extreme events. Our results indicate that combining multi-sensor satellite remote sensing data has the potential to support the monitoring of different proxies of coastal dynamics by overcoming the limitations of the individual sensor-types. Especially in coastal environments, where in-situ data with high temporal resolution is not available, integrating satellite remote sensing data can make a significant contribution for local coastal protection authorities.

## Material and methods

### Data

We jointly analyzed HR optical PlanetScope, MR optical Sentinel-2 and Landsat-8/9, and VHR TerraSAR-X Staring Spotlight SAR data to analyze different proxies of coastal dynamics at the Island of Langeoog. A time series from 2018 to 2023 is created from multi-sensor satellite data to ensure the densest possible temporal coverage of the entire period. In order to ensure comparability and optimal visibility of the coastal areas temporally flooded during high tide, only imagery that depicts the condition +/- 1.5 hours around low tide is selected regarding optical data. Table 7 provides an overview of the datasets used for this study.

**Table 7:** Datasets used.

Dataset	Acquisition Date	Scene Count	Spatial Resolution
PlanetScope	April 2018 - July 2023	16	3 m
Sentinel-2	January 2018 - September 2023	33	10-20 m
Landsat-8	January 2018 - July 2023	16	30 m
Landsat-9	December 2021 - March 2022	3	30 m
TerraSAR-X Staring Spotlight	January 2023 - December 2023	20	1.5 m x 0.23 m

#### Optical high resolution imagery

Due to the location of the Island of Langeoog in the North Sea, the availability of usable optical data was limited not only by the comparability of the tide level, but also by locally increased cloud cover. Launched in June 2016, the PlanetScope satellite constellation^21^ currently consists of over 200 operating CubeSats and is constantly growing. It provides high spatial and temporal coverage of the entire Earth’s land surface. The CubeSats fly in a sun-synchronized orbit at altitudes between 475 and 525 km, with a joint return time of one day. Three sensor types are represented in the constellation, which differ in terms of their technical specifications (e.g. their spatial resolution and the number of available spectral channels). The available data products are resampled to a spatial resolution of 3 m. The PlanetScope dataset used in this study consists of 16 L3B Ortho Tile Products, acquired between April 2018 and July 2023 (Table 7).

#### Optical mid resolution imagery

In order to condense the time series of multispectral data, we supplemented it with data acquired by the MR Landsat and Sentinel-2 satellite missions. Here as well, only data with very low cloud cover were selected. There are 33 acquisitions available from Sentinel-2 in the time period January 2018 to September 2023. The Sentinel-2^44^ satellite constellation is part of the European Union’s Copernicus programme. Sentinel-2A was launched in June 2015, followed by Sentinel-2B in March 2017. The sensors are in a sun-synchronized orbit at an altitude of 786 km and have a return time of five days. On board is the MSI (Multispectral Instrument); it covers 13 spectral channels in the VIS to SWIR range with geometric resolutions of 10/ 20/ 60 m.

Landsat-8^45^, launched in February 2013, orbits the Earth in a sun-synchronous track at an altitude of 705 km. On board is the OLI (Operational Land Imager) instrument, which provides images in nine spectral channels. These data have a spatial resolution of 15 m in panchromatic mode and 30 m in the visible to short wave infrared range (VIS-SWIR), with a repetition rate of 16 days. Landsat-9^46^, its successor, was launched in September 2021 and shares many of the same technical features. It also operates in a sun-synchronous orbit, with the OLI-2 instrument providing similarly high-resolution images in the same spectral ranges. Together, the two satellites enable even denser temporal coverage and thus improved observation of environmental changes. For the analysis of Landsat data, Collection 2 Level 1 is used. A total of 16 Landsat 8 and 3 Landsat 9 acquisitions are available from January 2018 to July 2023.

#### Synthetic Aperture Radar (SAR) Data

The twin satellites TerraSAR-X and TanDEM-X^47^ of the German Aerospace Center (DLR) are operating since June 2007 and June 2010, respectively. They fly in close formation at an altitude of 514 km with a return time of 11 days. Both satellites carry an X-band SAR sensor that enables data takes in three different main modes. In the project context, Staring Spotlight acquisitions were tasked specifically for the western coast of Langeoog in the year 2023. This gives us a total of 20 VHR SAR images in single HH polarization with a resolution of 1.5 m (Ground Range Resolution) x 0.23 m (Azimuth Resolution). Data is not limited here to +/- 1.5 h around low tide in order to keep the data density as high as possible. For this reason, the different tide conditions must especially be considered with regard to the identification of sand bars and the instantaneous waterline.

### Methods

#### Analysis of the instantaneous waterline, dune toe detection and estimation of dry sand areas from HR optical satellite imagery

The PlanetScope acquisitions are processed in a python environment, using the scikit-learn^48^ library. HR PlanetScope data are downloaded as an L3B product^21^. This data product is already preprocessed comprehensively: it contains both radiometric and sensory corrections; the data is orthorectified and projected onto the UTM coordinate system. Therefore, only minor additional processing steps, like histogram matching and coregistration are performed. For extracting multiple coastal dynamic parameters, in the first step, we calculated the NDWI (Normalized Difference Water Index)^49^ for each scene. The index uses the spectral information of the Green and NIR (Near Infrared) channels, providing a pixel mask with negative values for areas with no water content and positive values representing water areas:

$$\begin{aligned} NDWI = \frac{(Green - NIR)}{(Green + NIR)} \end{aligned}$$

The precise threshold was identifid by using Otsu’s algorithm.^50^ The results were further used for generating a binary mask, extracting both sand bars and the island’s instantaneous waterline. Furthermore, by applying a histogram analysis and identifying local minima by using Otsu’s multimodal threshold analysis,^51^ sand areas were extracted from the index information for detecting the dune toe, addressed as the landward boundary of the sandy beach area. As it is not possible to differentiate between “Dry Sand” (permanently dry sandy areas) and “Wet Sand” (temporary flooded sandy areas) by index information alone, we additionally applied a Random Forest algorithm; it uses four channels (Red, Green, Blue and NIR) as input data. Additionally, we added the NDWI as input layer. Before classification, segmentation was performed. We tested the SLIC (Simple Linear Iterative Clustering)^52^ and the QUICKSHIFT^53^ segmentation algorithm. As SLIC algorithm showed better results, we continued our analysis with it. The classification algorithm was trained and tested with four classes, “*Water*”, “*Wet Sand*”, “*Dry Sand*”, and “*Other Land Cover*”. The results were used for a comparison with results from index analysis. Furthermore, we used findings for class “*Dry Sand*” to create a time series for analyzing the permanently dry beach area.

#### Analysis of the instantaneous waterline and dune toe detection from MR optical satellite imagery

Sentinel-2 and Landsat acquisitions were downloaded and processed with the python based shoreline extraction tool SAET^38^, based on the SHOREX^54^ algorithm. The SAET tool can be used to automatically search and download suitable Sentinel-2 and Landsat-8/-9 data and further extract shoreline information from it. Preprocessing steps are also performed by the tool, so that no further manual steps are necessary. For extracting the shoreline, there are three processing steps to be done: First, a water-land interface segmentation is performed, either by applying the clusterization technique k-means, or by calculating one out of a selection of water indices: The availability of radiometric information in the SWIR (Short-Wave Infrared) ranges enables the calculation of the MNDWI (Modified Normalized Difference Water Index)^55^ and the AWEI (Automated Water Extraction Index)^56^. MNDWI is a further development of the NDWI. Its calculation is quite similar to that of NDWI, with the NIR channel being replaced by the SWIR channel:

$$\begin{aligned} MNDWI = \frac{(Green - SWIR)}{(Green + SWIR)} \end{aligned}$$

The index is more robust to built-up land, vegetation and soil noise, which is often vulnerable to miss-classifications as water. For the AWEI, there are two sub-types available, which do (AWEIsh) or do not (AWEInsh) specifically take into account the miss-classification of shadows of water areas. As we use cloud free acquisitions with unshaded beach areas, we chose the AWEInsh for further investigation. It uses the green, NIR and SWIR bands for index calculation:

$$\begin{aligned} AWEInsh = 4 * (\rho _{Green} - \rho _{SWIR2}) - (0.25 * \rho _{NIR} + 2.75 * \rho _{SWIR1}) \end{aligned}$$

with $\rho$ as the reflectance value of spectral bands. There are three options available for the following thresholding: Setting the value to 0, applying Otsu^50^- or multi-Otsu^51^ method. For our test case, we found that the AWEInsh in combination with the multi-Otsu method provided best results. In the second processing step, the land-water mask is refined by removing all shoreline pixels lying outside beach areas, which are provided by the European Coastal Zone Dataset by Copernicus Land Monitoring Service^57^. In the third step, sub-pixel shoreline extraction is performed based on the SHOREX system. A kernel analysis for each pixel classified as shoreline is conducted; we found erosion mode (the binarized mask is corrected seawards) and a kernel size of 3x3 best fitting for our case study. By applying these settings, we extracted shoreline information for MR satellite images. For further investigation on MR data usability and comparison with HR data, we used the indices calculated with SAET and additionally processed NDWI results to perform similar index thresholding already tested with HR data. Availability of multiple water indices further enabled comparisons regarding suitability for extracting sand bars, instantaneous waterline information, the dune toe and dry sand areas by performing histogram analysis and thresholding.

#### Dune toe extraction from VHR SAR data

VHR SAR data are processed in ESA’s Sentinel Application Platform (SNAP) and ArcGIS Pro. SAR data from TerraSAR-X undergo several pre-processing steps. First, the data is radiometrically calibrated to obtain accurate brightness values. This is followed by a geometric ellipsoid correction. A speckle filter is also applied to minimize spectral noise. Then pixel intensity values are converted to backscattering coefficient values measured in decibels (dB). After preprocessing, we first performed a visual check on the data if there are acquisitions during low tide, as the data takes were collected independent of tide level. We found four acquisitions taken during low tide, and showing some sand flat patterns, but as HH-polarized SAR data is not that suitable for extracting land-water boundaries as single VV^4^- or dual^58^-polarized acquisitions, due to strong perturbations in the data, it was not possible to extract shoreline and sand flat information from it. For dune toe extraction, tide level and water speckle are not relevant, so we used SAR data to obtain VHR dune toe information for the year 2024. For analyzing the data, we defined a reference line for the dune toe for the first acquisition of 2024 and created a buffer zone (100 m) around it. Within that buffer zone, we performed Otsu^50^ threshold analysis and Canny^59^ filtering to extract the dune toe.

#### Validation of the results

Validation of our results was performed by cross-comparison of the different data-types analyzed, as in-situ measurements were not available for the study site. Additionally, for validating findings regarding sand bars, instantaneous waterline and dune toe position, we manually extracted reference data from Digital Orthophotos with a spatial resolution of 20 cm (DOP20) and compared our results with them. For that purpose, we calculated transects for the reference data to identify the distance to the extracted results. Then we calculated mean and standard deviation.

## Data Availability

Original satellite data are available via DLR (TerraSAR-X and RapidEye), PlanetLabs (Planet), ESA (Sentinel-2), and USGS (Landsat).
